# Predicting early neurological deterioration in acute branch atheromatous disease without reperfusion therapy: a machine learning model

**DOI:** 10.3389/fnins.2026.1846221

**Published:** 2026-06-10

**Authors:** Li Zeng, Wei He, Xiuxiu Lu, Liangbing Zhang

**Affiliations:** Department of Neurology, The First People’s Hospital of Anqing Affiliated to Anhui Medical University, Anqing, China

**Keywords:** branch atheromatous disease, early neurological deterioration, machine learning, model, prediction, scoring system, shap, XGBoost

## Abstract

**Background:**

Acute branch atheromatous disease (BAD) is one of the leading contributors to morbidity and disability in Asia, and early neurological deterioration (END) is common in affected patients. This study aimed to establish machine learning models to predict the risk of END in patients without reperfusion therapy.

**Methods:**

Patients with acute BAD who did not receive reperfusion therapy were retrospectively enrolled. Core predictive features were selected by LASSO regression with bootstrap stability assessment, and we used seven machine learning algorithms to build models. XGBoost was selected based on validation performance, nested cross-validation, and 1,000-iteration bootstrap validation. A spline logistic regression model served as the non-linear baseline. SHAP analysis was used to explain the model and develop a simple scoring system. Model discrimination was assessed using the area under the receiver operating characteristic curve (AUC), and clinical utility was evaluated using decision curve analysis (DCA).

**Results:**

A total of 369 patients were included in our research. We screened predictive factors with LASSO regression and ultimately identified five key variables. These included maximum infarct area, lactate dehydrogenase (LDH), number of infarct slices, admission systolic blood pressure (SBP), and neutrophil count. The XGBoost model achieved the best overall performance, with AUC of 0.927 in the training set and 0.846 in the validation set. Nested cross-validation yielded an unbiased AUC of 0.866 (95% CI: 0.817–0.925), and bootstrap validation produced a mean OOB AUC of 0.855 (95% CI: 0.760–0.941). The scoring system stratified patients into low (0–6 points), intermediate (7–13 points), and high (14–20 points) risk groups. DCA demonstrated favorable clinical utility. SHAP analysis also indicated that maximum infarct area and LDH were the top two predictors of END.

**Conclusion:**

An XGBoost-based prediction model and a simple scoring system, integrating maximum infarct area, LDH, number of infarct slices, admission SBP, and neutrophil count, provide reliable END risk prediction for acute BAD patients without reperfusion therapy.

## Introduction

Acute branch atheromatous disease (BAD) is a common subtype of ischemic stroke. It accounts for approximately 9.1%–18.3% of acute ischemic stroke in Asian populations, whereas its disability rate at discharge is 52.6% (Fujiwara et al., 2024; Li et al., 2025). A previous study showed that many patients with BAD develop progressive neurological decline, a change that strongly predicts worse long-term functional results (Liu Y. et al., 2024). Early neurological deterioration (END) is a serious adverse event in the acute phase of BAD, with an incidence of 17%–75% (Xu et al., 2025). It significantly increases adverse 3-month functional outcomes, severe disability and the risk of early mortality (Li S. et al., 2024). Therefore, END is a key factor that impacts BAD patients’ outcomes (Liu et al., 2025).

Clinical experience supports the use of intensive antithrombotic therapy, which can lower the likelihood of END in these patients. Research on argatroban combined with dual antiplatelet therapy has demonstrated that this regimen significantly decreases END occurrence and improves 90-day functional outcomes (Xu et al., 2025). Another multicenter prospective study indicated that using cilostazol combined with antiplatelet therapy in the ultra-early stage might provide a novel option for preventing the clinical progression of BAD (Kimura et al., 2016). Additionally, the efficacy of tirofiban combined with aspirin is currently under evaluation in an ongoing multicenter randomized controlled trial (Liao et al., 2024). Nevertheless, given the potential bleeding risks of intensified treatment, developing a reliable method to identify high-risk populations is essential for enabling clinicians to provide targeted therapy to those most likely to benefit.

Previous studies have attempted to develop predictive models for END in BAD patients, yet substantial limitations remain. First, the study populations lacked adequate specificity. While some studies included patients receiving reperfusion therapy and others built machine learning models using large cohorts, both failed to rule out the confounding effects of reperfusion treatment (Wu et al., 2020; Jiang et al., 2024; Liu W. et al., 2024). It is worth noting that reperfusion therapy itself substantially alters the pathophysiology and risk profile of END. The Safe Implementation of Treatments in Stroke (SITS) registry reported an END incidence of 6.7% following intravenous thrombolysis, with symptomatic intracranial hemorrhage as the primary contributing factor (Yu et al., 2020). Consequently, models derived from such mixed populations cannot be directly applied to BAD patients without reperfusion therapy. Similarly, predictive models developed for the general acute ischemic stroke population are difficult to generalize to this specific subtype (Li N. et al., 2024). Second, methodological limitations persist in model development. Several studies relied primarily on traditional logistic regression and other linear approaches (Hoshino et al., 2025; Liu et al., 2025), which may inadequately capture the complex non-linear relationships between clinical characteristics and END occurrence.

Machine learning (ML), a core branch of artificial intelligence, can automatically learn patterns from data to make subsequent predictions (Ngiam and Khor, 2019). Compared with traditional linear models, it shows substantial advantages in capturing non-linear relationships and interactions among variables (Oh et al., 2025). In the field of cerebrovascular diseases, ML has been successfully applied to key tasks, including predicting imaging changes (Phillips et al., 2025), classifying stroke subtypes (Chen et al., 2024), and evaluating clinical prognosis (Heo et al., 2019), which provides a solid methodological foundation for the present study.

To the best of our knowledge, this is the first study to develop a prediction model for END in BAD patients without reperfusion therapy. In clinical practice, the majority of BAD patients miss the therapeutic time window or have contraindications, which leaves them unable to receive reperfusion treatment and ultimately results in their receiving only conservative medical management. We built the model exclusively in this subgroup, avoiding pathophysiological confounding and yielding more stable prediction results. At the same time, we used clinical and laboratory indicators routinely obtained at admission to build and validate a machine learning model, combined machine learning with SHAP interpretability, and translated SHAP values into a clinically usable scoring system. To our knowledge, this combination of strict cohort specificity and SHAP-based bedside transformation has not been reported in the END prediction of BAD before. This study combines prediction accuracy, interpretability, and practical clinical utility, enabling neurologists to make individualized antithrombotic intensity decisions for this common but currently underserved subgroup.

## Materials and methods

### Patient selection

This study was designed as a retrospective cohort study. Consecutive patients with acute BAD without reperfusion therapy who were admitted to Anqing First People’s Hospital between January 2022 and August 2025 were enrolled. The study was approved by the Ethics Committee of Anqing First People’s Hospital (approval number: AQYY-YXLL-LWLL-2608) and conducted in accordance with the revised Declaration of Helsinki. Written informed consent was waived due to the retrospective design. All patient information was de-identified during analysis. Patients were included if they met the following criteria: admission within 48 h of symptom onset; completion of neuroimaging within 48 h of admission with confirmed diagnosis of BAD; and without reperfusion therapy, including intravenous thrombolysis, interventional procedures, or surgical treatment. Exclusion criteria were as follows: intracranial hemorrhage on head CT; stenosis of ≥50% in the parent artery (basilar artery or middle cerebral artery) on vascular assessment (MRA, CTA, or DSA); and incomplete clinical data.

### Data acquisition

Demographic and clinical data included age, sex, height, weight, medical history (hypertension, coronary heart disease, diabetes mellitus, and previous stroke), smoking (current or previous), drinking, admission systolic blood pressure (SBP), admission diastolic blood pressure (DBP), admission NIHSS score, admission mRS score, onset to door time, and onset to first MRI time. Laboratory parameters comprised routine blood tests, admission blood glucose, glycated hemoglobin (HbA1c), uric acid, serum creatinine, blood urea nitrogen, estimated glomerular filtration rate (eGFR), total cholesterol, triglycerides, low-density lipoprotein (LDL), high-density lipoprotein (HDL), lactate dehydrogenase (LDH), albumin, homocysteine, D-dimer, and fibrinogen. Composite indices included monocyte to lymphocyte ratio (MLR), lymphocyte to monocyte ratio (LMR), neutrophil to monocyte to lymphocyte ratio (NMLR), systemic inflammatory response index (SIRI), non-high density lipoprotein cholesterol to high density lipoprotein cholesterol ratio (NHHR), and white blood cell to high density lipoprotein cholesterol ratio (WHR). All indices were calculated based on corresponding blood cell counts and lipid profiles. Imaging characteristics comprised infarct location (basal ganglia, internal capsule, thalamus, pons, lateral ventricle, centrum semiovale), maximum infarct area (calculated as the product of the longest diameter and perpendicular width), number of infarct slices, and stroke side. Measurements on diffusion-weighted imaging (DWI) sequences were performed independently by two neurologists, with discrepancies resolved through consensus discussion.

### Sample size

To ensure the robustness and generalizability of the predictive model, sample size estimation followed the events per variable (EPV) principle (Peduzzi et al., 1996). A total of 66 outcome events were observed, and LASSO regression retained five core predictors, yielding an EPV of 13.2 (66/5), which met the widely accepted criterion of EPV ≥ 10 for model development.

### Outcome definition

Early neurological deterioration served as the primary outcome measure, which was defined as an increase of ≥2 points on the total NIHSS or ≥1 point on its motor subscale during the first 7 days after hospital admission, compared with baseline assessment (Liu et al., 2023b).

### Data preprocessing

Before exclusion, 19 patients had incomplete data for other candidate variables such as lipid profiles, homocysteine, and HbA1c. The missing rate for each candidate variable was below 5%. Because the missing proportion was low and the core model variables were complete, we used complete-case analysis without multiple imputation. The study dataset included 303 patients without END and 66 with END. After stratification, patients were randomly assigned to the training and validation sets at a 7:3 ratio to ensure balanced outcome distribution between sets. Continuous variables in the training set were normalized using Min-Max scaling. We used Min-Max scaling to put all continuous features into the 0–1 range. The continuous variables in our data had different units and scales. For example, admission SBP was in mmHg, while neutrophil count was in ×10^9^/L. Min-Max scaling stops variables with larger values from taking over the model. All clinical indicators in this study were non-negative, and their normal ranges are well-defined in clinical practice. So Min-Max scaling is easier to follow than Z-score standardization, which can give negative numbers. We did not use RobustScaler because our clinical variables have clear normal ranges, and extreme values do not often appear. Least Absolute Shrinkage and Selection Operator (LASSO) regression (α = 1) combined with 10-fold cross-validation was applied to the training set to select predictive variables. The validation set was not involved in feature selection or model training and was used only for final performance evaluation. Ultimately, five variables were identified: maximum infarct area, LDH, number of infarct slices, admission SBP, and neutrophil count. To assess the stability of LASSO feature selection, we performed 1,000 bootstrap resampling iterations on the training set and recorded the selection frequency of each candidate variable.

### Machine learning algorithms

Based on the five selected features, seven machine learning prediction models were developed using Python 3.10.4, including logistic regression, decision tree, random forest (RF), extreme gradient boosting (XGBoost), light gradient boosting machine (LightGBM), support vector machine (SVM), and artificial neural network (ANN). Grid search and 5-fold cross-validation were used to fine-tune our model’s hyperparameters. The final parameter configurations were as follows: logistic regression (default settings, including a constant term); decision tree (max_depth = 10, min_samples_split = 20); random forest (n_estimators = 20, max_features = 2); XGBoost (n_estimators = 180, max_depth = 3, learning_rate = 0.03, subsample = 0.65, colsample_bytree = 0.55, min_child_weight = 3, reg_alpha = 0.5, reg_lambda = 7); LightGBM (n_estimators = 150, num_leaves = 7, learning_rate = 0.03, subsample = 0.5, colsample_bytree = 0.6, min_child_samples = 20, reg_alpha = 5, reg_lambda = 5); SVM (kernel = ‘linear’, C = 0.1, gamma = ‘scale’); and ANN (hidden_layer_sizes = (100, 100), activation = ‘logistic’). Strong regularization parameters (e.g., reg_alpha, reg_lambda, shallow tree depths) were deliberately chosen to mitigate overfitting given our limited sample size. A linear kernel was used for SVM to maintain interpretability with only five features, and a moderate two-layer architecture was selected for ANN to balance model capacity and overfitting risk. To benchmark against simpler interpretable models, we fitted a logistic regression model with restricted cubic splines using three knots for each continuous variable. This spline model served as a non-linear baseline for comparison with XGBoost. We assessed model performance from discrimination, calibration and clinical utility. Discrimination was quantified using the AUC. A total of 95% confidence intervals were calculated using bootstrap resampling with 1,000 iterations. Calibration was assessed through calibration curves and the Brier score. Clinical utility was determined using decision curve analysis to evaluate net benefit. Accuracy, sensitivity, specificity, precision, and F1 score were calculated using the optimal threshold determined by the Youden index for each model.

### Nested cross-validation

In order to achieve more robust model evaluation and effectively avoid overfitting, this study performed 5 × 4 nested cross-validation for all seven candidate models. Enhanced regularization was applied during hyperparameter tuning. The outer loop split the data into 5-folds for unbiased model evaluation. The inner loop used 4-fold cross-validation combined with grid search to optimize regularization hyperparameters. This design ensured that hyperparameter tuning and model performance evaluation remained independent, preventing information leakage.

### Bootstrap internal validation

To assess model generalizability and correct for optimism bias, we performed strict out-of-bag (OOB) bootstrap validation with 1,000 iterations for the XGBoost model. In each iteration, a bootstrap sample of size *n* = 260 was drawn with replacement from the full cohort; the unsampled observations served as the independent validation set. The in-sample AUC and OOB AUC were computed for each iteration, with optimism defined as in-sample AUC minus OOB AUC. The mean OOB AUC and its 95% confidence interval were reported as the primary bias-corrected estimate. Iterations yielding fewer than 20 OOB samples or fewer than five positive events in the OOB set were excluded from summary statistics.

### Development of a scoring system

This study, based on SHAP analysis results, transformed the XGBoost model into a simple scoring system. The points for each variable were decided by its SHAP contribution. For the cutoff values, we checked the change patterns in SHAP dependence plots and also used the Youden index from single-variable ROC analysis, then rounded these to whole numbers that doctors can easily remember.

### SHAP interpretability analysis

The optimal model was interpreted post-hoc using the SHAP (SHapley Additive exPlanations) framework (Lundberg et al., 2020). Visual analyses were conducted to illustrate the magnitude and direction of each input feature’s contribution to the predicted risk of END.

### Statistical analysis

Baseline statistical analyses were performed using R version 4.5.1. Continuous variables were assessed for normality using the Shapiro-Wilk test. Normally distributed variables were described as mean (standard deviation, SD) and compared between groups using independent samples *t*-test. Non-normally distributed variables were presented as median (interquartile range, IQR) and compared using Wilcoxon rank-sum test. Categorical variables were expressed as *n* (%) and compared using the χ^2^ test. The “CreateTableOne” function was used to generate baseline comparison tables between training and validation sets. A *P*-value < 0.05 is considered statistically significant.

## Results

**FIGURE 1:**
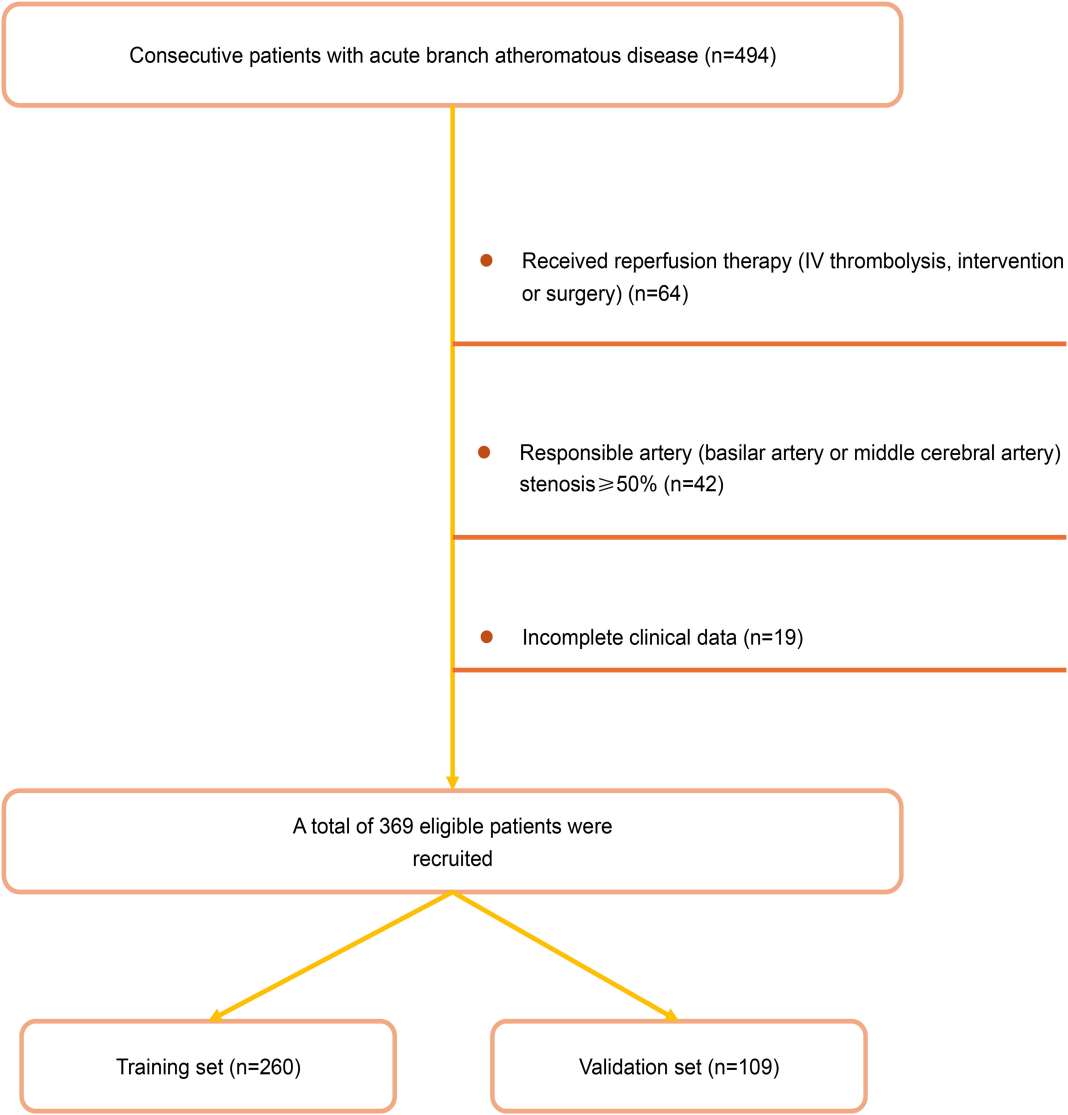
Flow diagram of the included patients.

**FIGURE 2:**
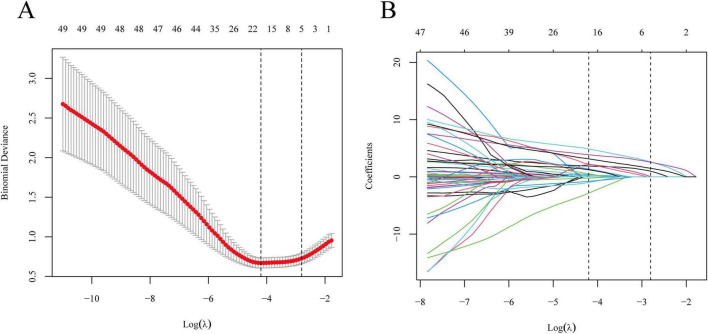
Predictor selection using the Least Absolute Shrinkage and Selection Operator (LASSO) regression analysis with 10-fold cross-validation. **(A)** The cross-validated binomial deviance curve of the LASSO model is plotted against log(λ). Red dots represent the mean binomial deviance, with gray error bars indicating standard errors. Vertical dashed lines mark the optimal λ values. The value of λ = 0.015 corresponds to the minimum deviance and λ = 0.061 refers to the one-standard-error criterion. **(B)** The coefficient profiles of all candidate predictors are shown across the log((λ) sequence, with vertical dashed lines aligned to the corresponding optimal λ values. According to the one-standard-error rule, five variables with non-zero coefficients were retained at λ = 0.061 for subsequent modeling.

**FIGURE 3:**
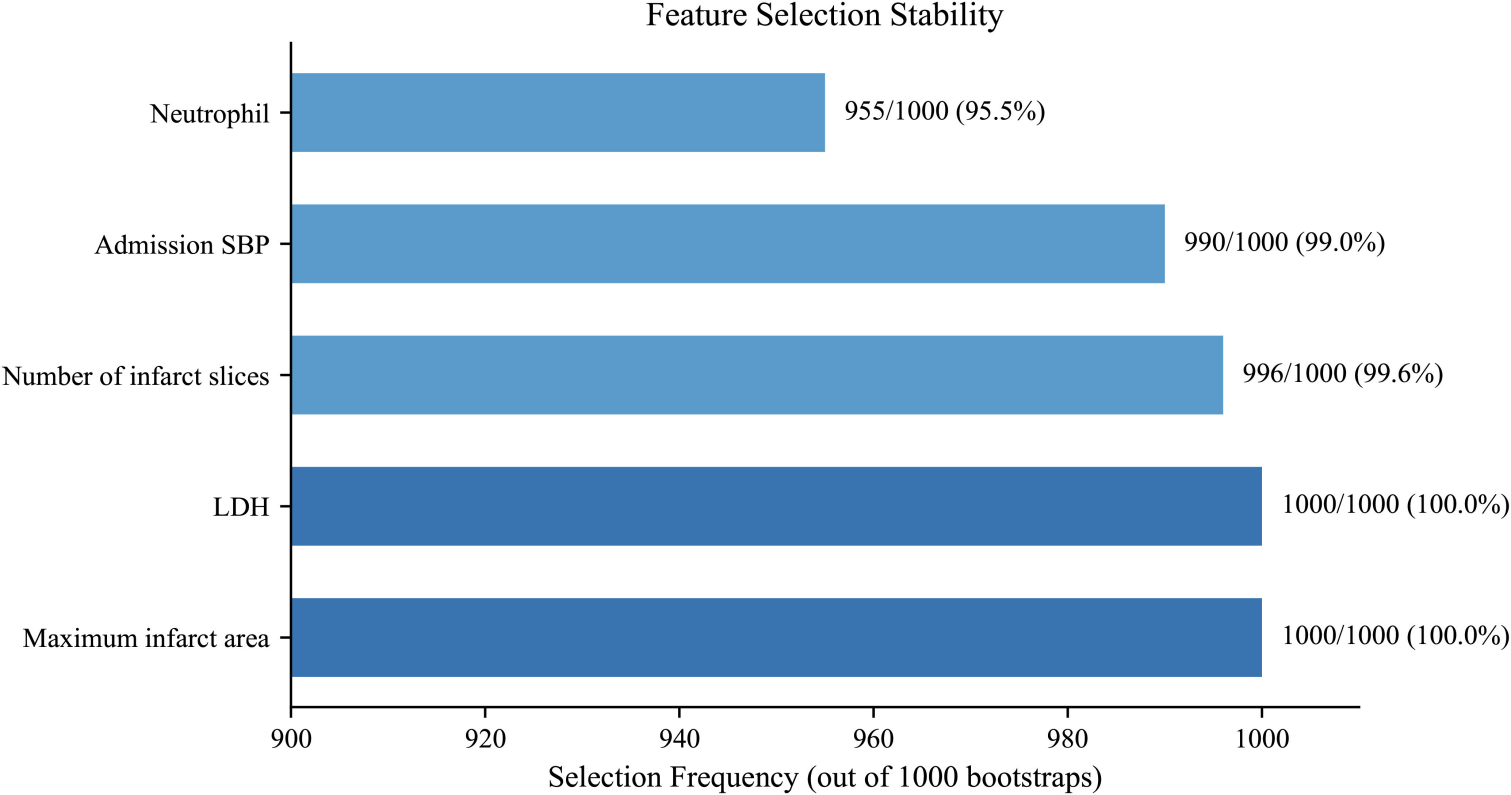
Stability of Least Absolute Shrinkage and Selection Operator (LASSO) feature selection based on 1,000 bootstrap resampling iterations on the training set. Bars represent the selection frequency of each variable across 1,000 bootstrap samples. Values in parentheses indicate the percentage of iterations in which the variable was selected.

A flowchart illustrating the participant selection process is shown in Figure 1. After excluding patients with reperfusion therapy, responsible artery stenosis ≥ 50%, or incomplete data, 369 patients were included in the final analysis. The average age of the cohort was 67 (range 58–75) years, with 207 males (56.1%) and 162 females (43.9%) (Table 1; detailed baseline comparisons including composite inflammatory indices and other laboratory parameters are provided in Supplementary Table 1). All participants were randomly assigned to the training set (*n* = 260) and validation set (*n* = 109) at a 7:3 ratio. The comparison of baseline clinical characteristics between the two sets of patients is presented in Table 2 (full data are available in Supplementary Table 2). Statistical analysis revealed that there were no significant differences between the two sets in all included variables (*p* > 0.05), and the prevalence of END was comparable in both sets (18.1% vs. 17.4%, *P* > 0.99). We used LASSO regression to select variables on the training set (λ_1*se*_ = 0.061) (Figure 2), and finally included five core features, namely, maximum infarct area, LDH, number of infarct slices, admission SBP, and neutrophil count. Figure 3 shows the stability of LASSO feature selection. Maximum infarct area and LDH were selected in all 1,000 bootstrap samples (100%), followed by number of infarct slices (99.6%), admission SBP (99.0%), and neutrophil count (95.5%). Multivariate logistic regression analysis, as shown in Table 3, showed that the above five characteristics were independent influencing factors for predicting END.

**TABLE 1:** Comparing baseline characteristics between early neurological deterioration (END) and Non-END groups in patients.

Baseline characteristics	Overall (*n* = 369)	Non-END (*n* = 303)	END (*n* = 66)	*P*
Demographic data				
Age (years), median (IQR)	67.00 [58.00, 75.00]	67.00 [58.00, 75.00]	63.00 [56.00, 76.00]	0.761
Male, *n* (%)	207 (56.1)	174 (57.4)	33 (50.0)	0.335
Height (cm), median (IQR)	165.00 [158.00, 170.00]	165.00 [158.00, 170.00]	163.50 [160.00, 170.00]	0.733
Weight (kg), median (IQR)	65.00 [55.00, 71.00]	65.00 [55.50, 71.00]	65.00 [55.00, 72.00]	0.949
SBP (mmHg), median (IQR)	152.00 [139.00, 166.00]	150.00 [136.50, 165.50]	161.00 [152.25, 177.00]	<0.001*
DBP (mmHg), median (IQR)	86.00 [79.00, 98.00]	86.00 [78.00, 96.00]	94.50 [85.00, 102.00]	0.001*
Medical history, n (%)				
Hypertension	265 (71.8)	213 (70.3)	52 (78.8)	0.216
Diabetes mellitus	67 (18.2)	54 (17.8)	13 (19.7)	0.856
Coronary heart disease	32 (8.7)	23 (7.6)	9 (13.6)	0.180
Previous stroke	56 (15.2)	45 (14.9)	11 (16.7)	0.855
Smoking current or previous	123 (33.3)	101 (33.3)	22 (33.3)	1.000
Drinking	80 (21.7)	67 (22.1)	13 (19.7)	0.79
Clinical features				
Admission NIHSS score, median (IQR)	3.00 [2.00, 4.00]	3.00 [2.00, 4.00]	4.00 [3.00, 5.00]	<0.001*
Admission mRS score, median (IQR)	3.00 [1.00, 4.00]	2.00 [1.00, 3.00]	3.00 [3.00, 4.00]	<0.001*
Laboratory data				
Leukocytes (×10^9^/L), median (IQR)	6.25 [5.16, 7.32]	6.14 [5.04, 7.22]	6.79 [5.79, 8.04]	0.001*
Neutrophil (×10^9^/L), median (IQR)	3.93 [3.12, 4.93]	3.80 [2.95, 4.70]	4.66 [3.81, 5.34]	<0.001*
Lymphocyte (×10^9^/L), median (IQR)	1.51 [1.13, 1.97]	1.51 [1.10, 1.96]	1.52 [1.20, 2.06]	0.494
Monocyte (×10^9^/L), median (IQR)	0.41 [0.32, 0.53]	0.42 [0.33, 0.52]	0.41 [0.31, 0.53]	0.586
Admission blood glucose (mmol/L), median (IQR)	5.85 [4.92, 7.56]	5.70 [4.89, 7.46]	6.85 [5.40, 8.10]	0.009*
HbA1c (%), median (IQR)	6.10 [5.50, 6.90]	5.90 [5.50, 6.60]	7.15 [6.50, 7.75]	<0.001*
Lactate dehydrogenase (U/L), median (IQR)	178.00 [156.00, 204.00]	174.00 [151.00, 198.00]	200.50 [178.50, 222.75]	<0.001*
Albumin (g/L), mean (SD)	41.18 (4.5)	40.88 (4.4)	42.53 (4.8)	0.007*
Homocysteine (μmol/L), median (IQR)	10.70 [8.80, 13.60]	10.50 [8.70, 13.15]	12.60 [9.50, 16.58]	0.004*
Maximum infarct area (mm^2^), median (IQR)	70.89 [48.53, 104.80]	63.40 [45.65, 94.57]	121.89 [81.07, 177.35]	<0.001*
Number of infarct slices, median (IQR)	2.00 [2.00, 3.00]	2.00 [2.00, 3.00]	3.00 [2.00, 4.00]	<0.001*

Values are presented as *n* (%), mean (SD), or median (interquartile range), as appropriate. Continuous variables were tested for normality by the Shapiro-Wilk test. Normally distributed data were compared using the independent samples *t*-test, and non-normally distributed data were analyzed using the Wilcoxon rank-sum test. Categorical variables were compared using the χ^2^ test. A *P*-value < 0.05 was considered statistically significant. SBP, systolic blood pressure; DBP, diastolic blood pressure; mRS, modified Rankin Scale; NIHSS, National Institutes of Health Stroke Score; HbA1c, glycated hemoglobin. *P < 0.05.

**TABLE 2:** Comparison of clinical data between the training set and validation set.

Baseline characteristics	Total (*n* = 369)	Training (*n* = 260)	Validation (*n* = 109)	*P*
END, *n* (%)	66 (17.9)	47 (18.1)	19 (17.4)	1.000
Demographic data				
Age (years), median (IQR)	67.00 [58.00, 75.00]	66.00 [57.00, 75.00]	67.00 [59.00, 74.00]	0.941
Male, *n* (%)	207 (56.1)	147 (56.5)	60 (55.1)	0.882
Height (cm), median (IQR)	165.00 [158.00, 170.00]	165.00 [158.00, 170.00]	165.00 [160.00, 170.00]	0.664
Weight (kg), median (IQR)	65.00 [55.00, 71.00]	65.00 [55.00, 71.25]	64.00 [55.00, 70.00]	0.295
SBP (mmHg), median (IQR)	152.00 [139.00, 166.00]	152.00 [139.75, 166.00]	155.00 [136.00, 168.00]	0.827
DBP (mmHg), median (IQR)	86.00 [79.00, 98.00]	86.00 [80.00, 96.00]	90.00 [78.00, 101.00]	0.864
Medical history, n (%)				
Hypertension	265 (71.8)	185 (71.2)	80 (73.4)	0.757
Diabetes mellitus	67 (18.2)	49 (18.9)	18 (16.5)	0.702
Coronary heart disease	32 (8.7)	23 (8.9)	9 (8.3)	1.000
Previous stroke	56 (15.2)	36 (13.9)	20 (18.4)	0.347
Smoking current or previous	123 (33.3)	91 (35.0)	32 (29.4)	0.353
Drinking	80 (21.7)	59 (22.7)	21 (19.3)	0.555
Clinical features				
Admission NIHSS score, median (IQR)	3.00 [2.00, 4.00]	3.00 [2.00, 4.00]	3.00 [2.00, 4.00]	0.449
Admission mRS score, median (IQR)	3.00 [1.00, 4.00]	3.00 [1.00, 4.00]	2.00 [1.00, 4.00]	0.580
Laboratory data				
Leukocytes (×10^9^/L), median (IQR)	6.25 [5.16, 7.32]	6.28 [5.16, 7.40]	6.23 [5.20, 7.24]	0.611
Neutrophil (×10^9^/L), median (IQR)	3.93 [3.12, 4.93]	3.98 [3.15, 4.94]	3.85 [3.11, 4.68]	0.592
Lymphocyte (×10^9^/L), median (IQR)	1.51 [1.13, 1.97]	1.50 [1.15, 1.94]	1.52 [1.11, 2.09]	0.698
Monocyte (×10^9^/L), median (IQR)	0.41 [0.32, 0.53]	0.42 [0.33, 0.53]	0.41 [0.31, 0.53]	0.632
Admission blood glucose (mmol/L), median (IQR)	5.85 [4.92, 7.56]	6.00 [4.94, 7.54]	5.53 [4.86, 7.56]	0.401
HbA1c (%), median (IQR)	6.10 [5.50, 6.90]	6.10 [5.60, 7.00]	6.00 [5.50, 6.90]	0.491
Lactate dehydrogenase (U/L), median (IQR)	178.00 [156.00, 204.00]	177.50 [155.00, 204.00]	180.00 [158.00, 204.00]	0.671
Albumin (g/L), mean (SD)	41.18 (4.5)	41.20 (4.6)	41.11 (4.1)	0.857
Homocysteine (μmol/L), median (IQR)	10.70 [8.80, 13.60]	10.80 [8.80, 13.62]	10.20 [8.70, 13.60]	0.496
Maximum infarct area (mm^2^), median (IQR)	70.89 [48.53, 104.80]	70.12 [49.75, 105.74]	73.94 [47.22, 103.32]	0.543
Number of infarct slices, median (IQR)	2.00 [2.00, 3.00]	2.00 [2.00, 3.00]	2.00 [2.00, 3.00]	0.941

Values are presented as *n* (%), mean (SD), or median (interquartile range), as appropriate. Continuous variables were tested for normality by the Shapiro-Wilk test. Normally distributed data were compared using the independent samples *t*-test, and non-normally distributed data were analyzed using the Wilcoxon rank-sum test. Categorical variables were compared using the χ^2^ test. END, early neurological deterioration; SBP, systolic blood pressure; DBP, diastolic blood pressure; mRS, modified Rankin Scale; NIHSS, National Institutes of Health Stroke Score; HbA1c, glycated hemoglobin.

**TABLE 3:** Multivariate logistic regression.

Variable	OR	95%CI	*P*
Admission SBP	1.03	(1.01, 1.05)	0.010
Neutrophil	1.29	(1.03, 1.67)	0.035
LDH	1.03	(1.01, 1.04)	<0.001
Maximum infarct area	1.02	(1.01, 1.03)	<0.001
Number of infarct slices	2.09	(1.28, 3.55)	0.004

CI, confidence interval; OR, odds ratio.

**FIGURE 4:**
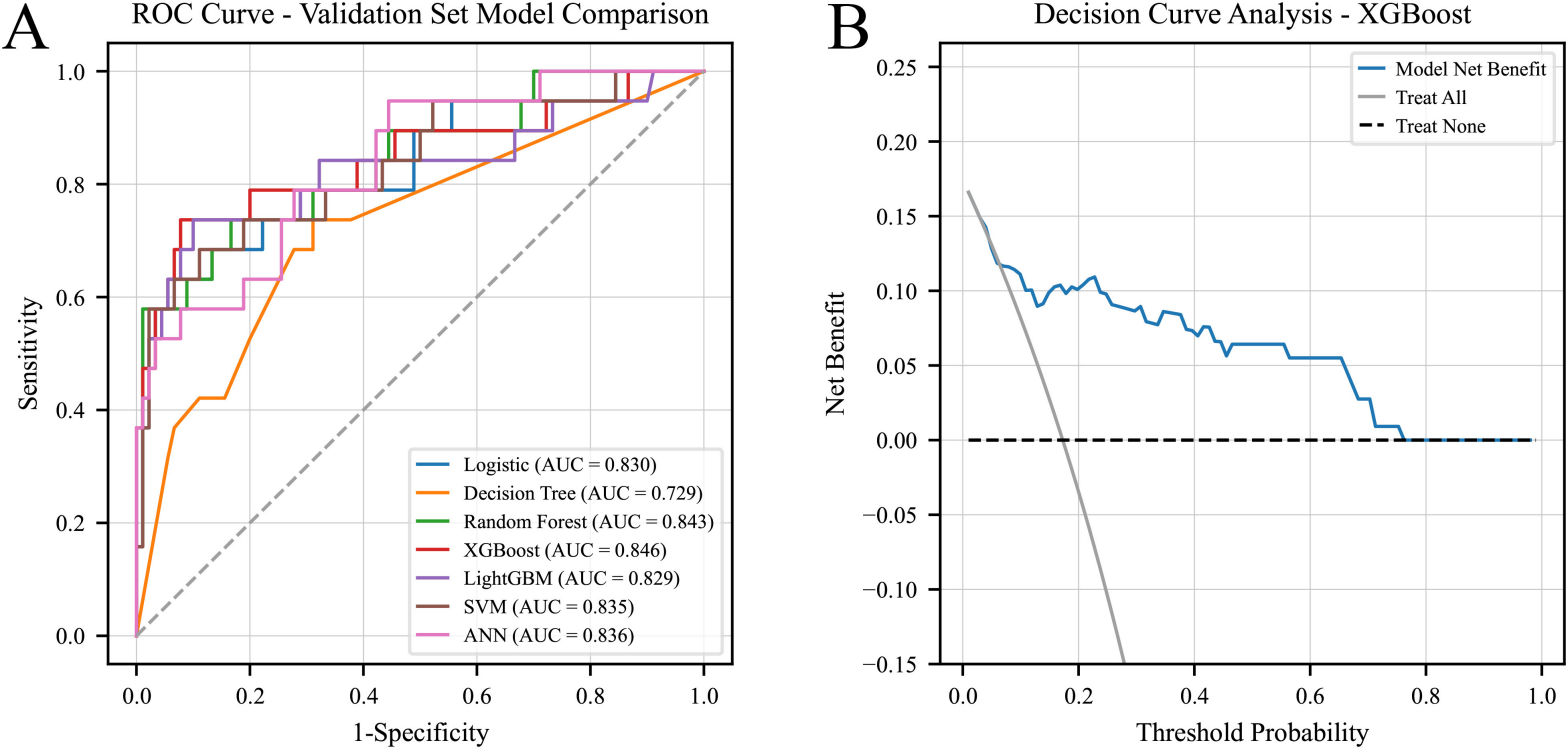
**(A)** ROC curves of seven machine learning algorithms based on the validation set. **(B)** Decision curve analysis (DCA) of the extreme gradient boosting (XGBoost) machine learning model in the validation set. **(A)** The x-axis represents 1-Specificity (false positive rate), and the y-axis represents Sensitivity (true positive rate). The ROC curves of seven models, including Logistic regression, Decision Tree, Random Forest, XGBoost, LightGBM, support vector machine (SVM) and artificial neural network (ANN), are presented, with area under the receiver operating characteristic curve (AUC) of each model labeled in parentheses. **(B)** The x-axis denotes the threshold probability, while the y-axis denotes the net benefit. The black dashed line represents the strategy of “treat none” (net benefit = 0). The gray solid line represents the strategy of “treat all” patients. The blue line represents the net benefit derived from the machine learning model.

Figure 4A and Table 4 present the performance of the seven models in the validation set. The XGBoost model showed the best overall performance, with an AUC of 0.846 and an accuracy of 0.890. The random forest model ranked second with an AUC of 0.843, but its specificity (0.833 vs. 0.922) and precision (0.483 vs. 0.667) were both lower than those of XGBoost. Logistic regression had the highest specificity (0.944) and precision (0.706), yet its AUC (0.830) and sensitivity (0.632) were not as good as those of XGBoost. Also, XGBoost gave the lowest Brier score at 0.090 (95% CI: 0.054–0.130). Its calibration curve also showed a small overall calibration error, suggesting good agreement between predicted risks and actual outcomes (Supplementary Figure 1). Decision curve analysis (DCA) showed that the XGBoost model had good clinical usefulness across a wide threshold range of 0.04–0.76, with net benefits over both the “treat all” and “treat none” strategies (Figure 4B). Using the Youden index, the best probability cutoff was 0.236. At this cutoff, the model gave a sensitivity of 0.737 and a specificity of 0.922.

**TABLE 4:** Prediction results of seven machine learning algorithms based on the validation set.

Model	AUC	95% CI lower	95% CI upper	Accuracy	Precision	Sensitivity	Specificity	F1 score	Kappa	Youden’s J	PPV	NPV
Logistic	0.830	0.707	0.933	0.890	0.706	0.632	0.944	0.667	0.601	0.576	0.706	0.924
Decision tree	0.729	0.590	0.860	0.697	0.333	0.737	0.689	0.459	0.288	0.426	0.333	0.925
Random forest	0.843	0.720	0.942	0.817	0.483	0.737	0.833	0.583	0.472	0.570	0.483	0.938
XGBoost	0.846	0.718	0.953	0.890	0.667	0.737	0.922	0.700	0.633	0.659	0.667	0.943
LightGBM	0.829	0.683	0.945	0.872	0.609	0.737	0.900	0.667	0.588	0.637	0.609	0.942
SVM	0.835	0.715	0.938	0.853	0.565	0.684	0.889	0.619	0.529	0.573	0.565	0.930
ANN	0.836	0.714	0.927	0.734	0.375	0.789	0.722	0.508	0.356	0.512	0.375	0.942

AUC, area under the curve; CI, confidence interval; PPV, positive predictive value; NPV, negative predictive value.

Multiple validation methods all confirmed that XGBoost was robust. The gap in AUC between the training set and the validation set was 0.081. The nested cross validation gave an unbiased AUC of 0.866 (95% CI: 0.817–0.925). Bootstrap internal validation with 1,000 iterations produced a mean OOB AUC of 0.855 (95% CI: 0.760–0.941). Both results were very close to the main analysis validation set AUC of 0.846 (Supplementary Tables 3–5).

We used restricted cubic spline logistic regression as the baseline model. On the validation set, XGBoost outperformed the spline logistic regression baseline in AUC (0.846 vs. 0.807), Brier score (0.089 vs. 0.103), and sensitivity (0.737 vs. 0.474). DCA also showed that XGBoost gave higher net benefit (Supplementary Figure 2). Although the DeLong test did not reach statistical significance (*Z* = 1.913, *P* = 0.056), the consistent advantages in calibration and clinical usefulness supported choosing XGBoost.

**FIGURE 5:**
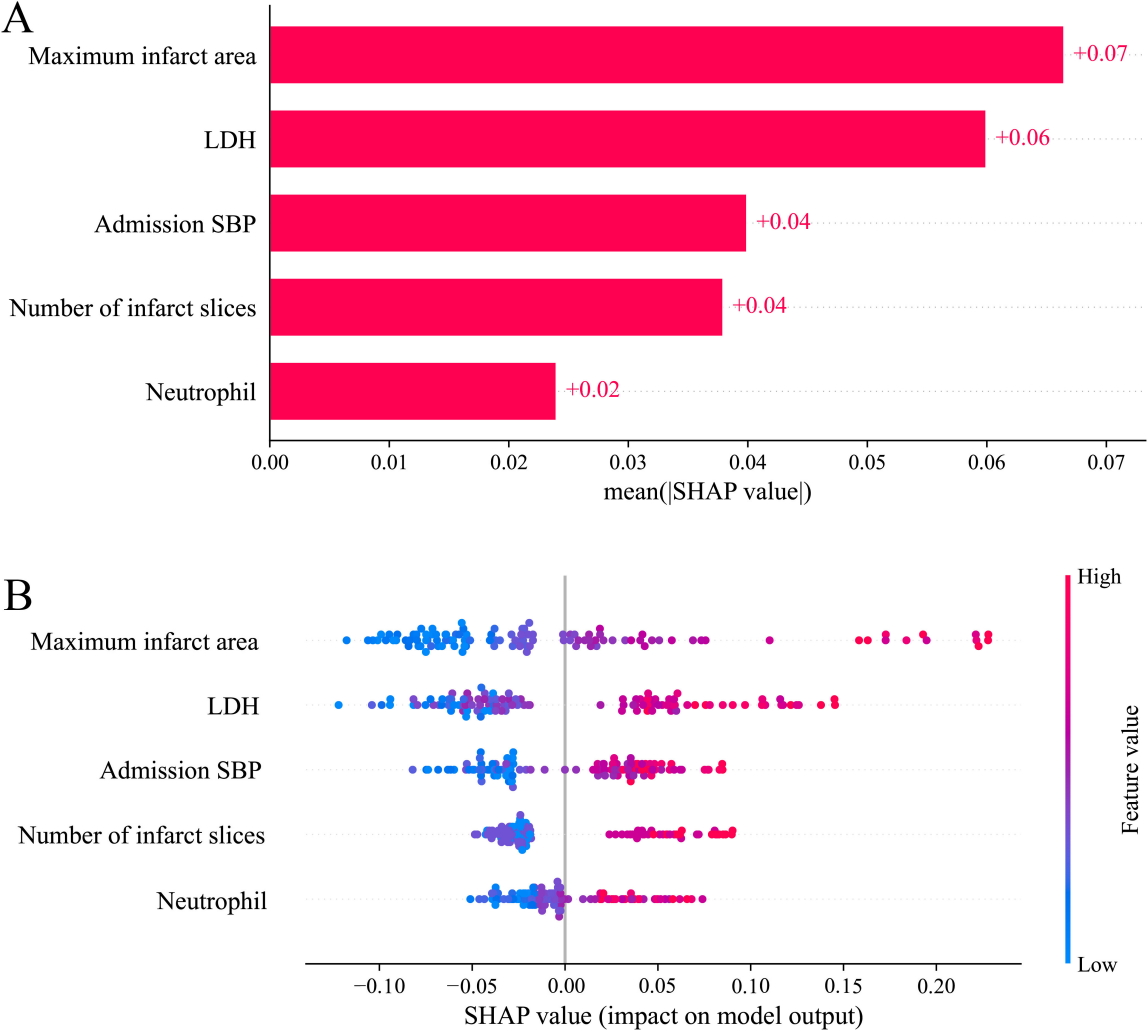
SHapley Additive exPlanations (SHAP) analysis of the extreme gradient boosting (XGBoost) machine learning model. **(A)** Summary bar plot of mean absolute SHAP values, ranking the five predictive features by their overall impact on model output. **(B)** Beeswarm plot of SHAP values, illustrating the direction and magnitude of each feature’s effect on the prediction of early neurological deterioration (END). Each dot represents one patient. The horizontal position indicates the SHAP value (positive values increase END risk, negative values decrease risk). The color represents the actual feature value, ranging from blue (low) to red (high). For features such as maximum infarct area, lactate dehydrogenase (LDH), and number of infarct slices, higher feature values are associated with positive SHAP values (increased risk), while red dots cluster on the right side of the plot. A wider spread of SHAP values indicates greater heterogeneity in the feature’s impact across patients.

**FIGURE 6:**
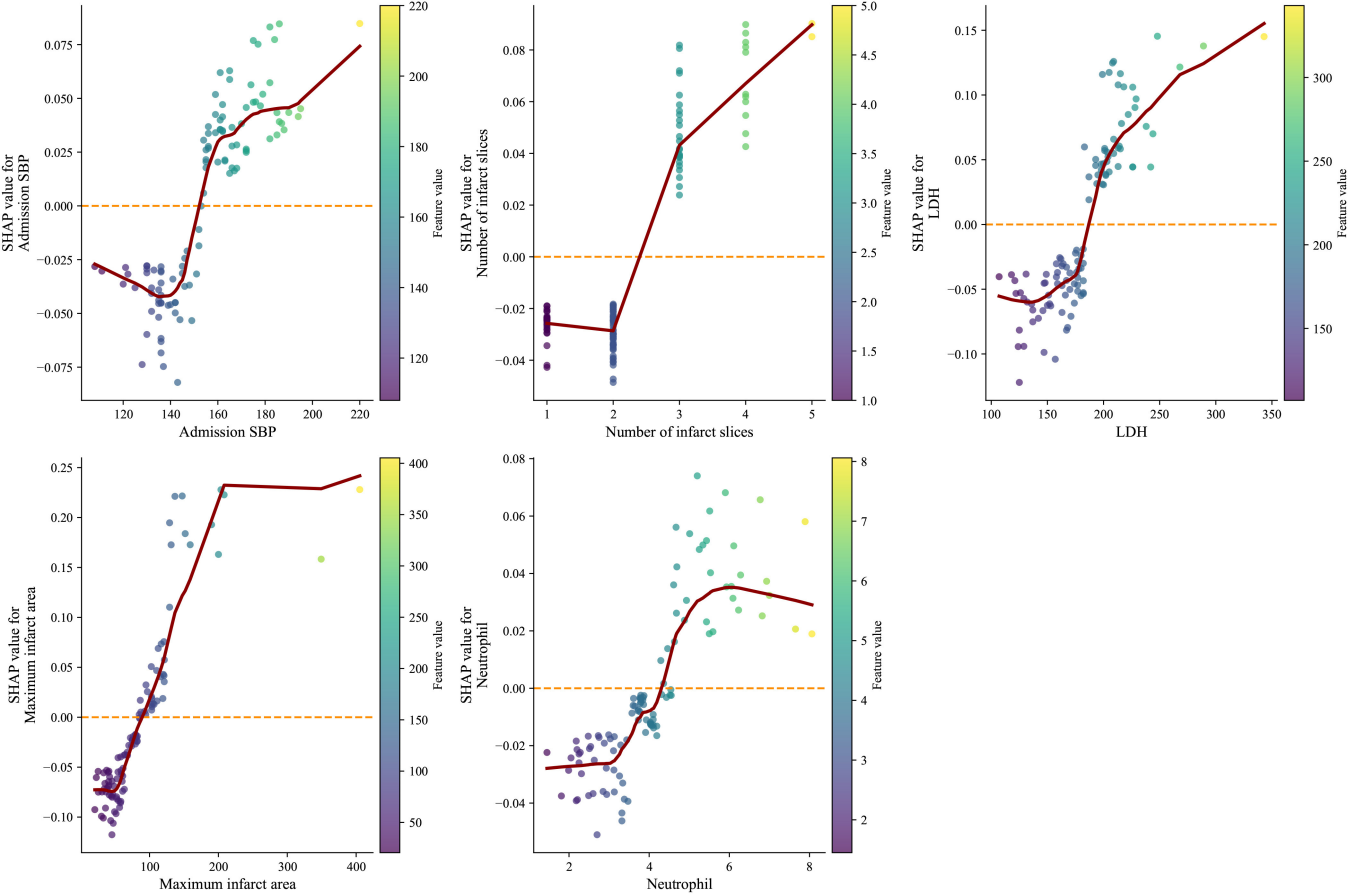
SHapley Additive exPlanations (SHAP) dependence plots of the extreme gradient boosting (XGBoost) machine learning model. SHAP dependence plots for the five predictive features: maximum infarct area, lactate dehydrogenase (LDH), number of infarct slices, admission systolic blood pressure (SBP), and neutrophil count. Each plot illustrates the relationship between the feature value (color-coded from low to high) and its corresponding SHAP value (impact on model output), with a brown line indicating the locally estimated scatterplot smoothing (LOESS) trend. Positive SHAP values indicate increased early neurological deterioration (END) risk, while negative values indicate decreased risk.

We used SHAP analysis to explain the prediction basis of the best XGBoost model and to improve its clinical interpretability. The SHAP bar plot (Figure 5A) ranked that maximum infarct area and LDH were the most important predictors, with mean absolute SHAP values of 0.07 and 0.06, respectively. Admission SBP and the number of infarct slices each had a mean absolute SHAP value of 0.04, and neutrophil count had a value of 0.02. The beeswarm plot (Figure 5B) showed how feature values drive predictions for individual patients. Each dot represents one patient. The horizontal position indicates the SHAP value, and the color shows the actual feature value, ranging from blue (low) to red (high). This plot displayed that higher values of maximum infarct area, LDH, and number of infarct slices were linked to a higher risk of END. The SHAP dependence plots (Figure 6) further revealed threshold effects. LDH had very little effect on risk below about 180 U/L, but after about 200 U/L its effect increased sharply, with SHAP values rising quickly. The admission SBP curve indicated a non-linear relationship. Risk was lowest at 130–140 mmHg and increased sharply after 140 mmHg. The curve increased more slowly between about 165 and 190 mmHg, but became steeper again after about 190 mmHg. The number of infarct slices showed a sharp threshold jump at three slices, where SHAP values turned from negative to positive. Maximum infarct area suggested a steady positive relationship. Neutrophil showed an S-shape with a relatively weak overall effect. It changed from protective to risk increasing at approximately 4.0 × 10^9^/L.

**FIGURE 7:**
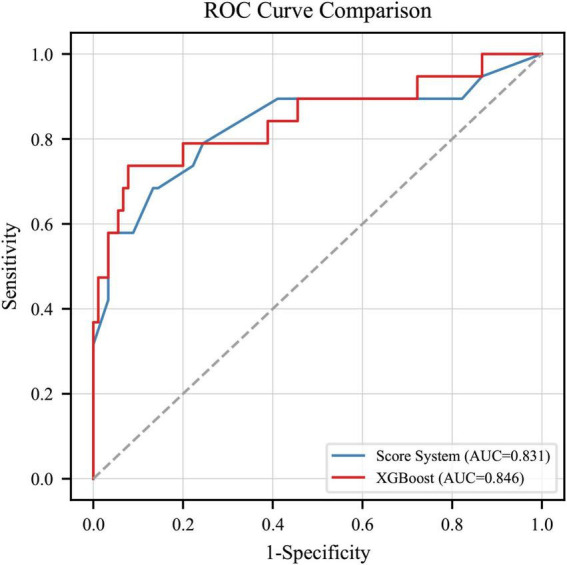
ROC curves comparison between the scoring system and the extreme gradient boosting (XGBoost) model in the validation set. The blue curve represents the scoring system, the red curve represents the XGBoost model, and the diagonal dashed line indicates the reference line.

To improve bedside clinical usefulness, we turned the XGBoost model into a simple scoring system based on the SHAP analysis. The scoring system included five factors from the XGBoost model, with total scores ranging from 0 to 20 (Table 5). Patients were divided into three risk groups. The low risk group (scores 0–6) had an END rate of 4.17%, the medium risk group (scores 7–13) had an END rate of 12.77%, and the high risk group (scores 14–20) had an END rate of 78.57%. The AUC of this score in the validation set was 0.831, which was close to that of the XGBoost model (0.846) (Figure 7).

**TABLE 5:** Point-based scoring system for predicting early neurological deterioration (END) risk.

Variable	Score if	Points
Maximum infarct area	≥100 mm^2^	7
LDH	≥185 U/L	5
Number of infarct slices	≥3	3
Admission SBP	≥155 mmHg	3
Neutrophil	≥4.0 × 10^9^/L	2

Risk stratification based on total score: low risk (0–6 points, END rate 4.17%), intermediate risk (7–13 points, END rate 12.77%), high risk (14–20 points, END rate 78.57%). Variable cutoffs were determined by combining the inflection points identified from SHAP dependence plots with the Youden index from univariate ROC analysis, then rounded to clinically memorable integers. Points were assigned proportionally to SHAP contributions (7:5:3:3:2).

## Discussion

In this study, we employed a variety of machine learning models to identify the risk factors for END occurrence in patients with acute BAD without reperfusion therapy. Machine learning improved prediction accuracy by integrating multimodal data and capturing non-linear relationships, which has obvious advantages in predicting the prognosis of stroke (Akinwumi et al., 2025; Lv et al., 2025; Pu et al., 2026). Although there have been prediction models for predicting the END risk of BAD patients, previous studies did not exclude patients receiving reperfusion treatment. Moreover, reperfusion treatment itself can significantly affect the outcome of neurological function, which might lead to confounding bias. To solve this gap, we developed a prediction model expressly for acute BAD patients without reperfusion therapy, which can more accurately recognize risk factors related to the course of natural diseases. We used LASSO regression to determine five key predictive features, including maximum infarct area, LDH, number of infarct slices, admission SBP, and neutrophil count.

Among imaging indicators, the number of infarct slices and maximum infarct area directly reflect the extent of ischemic injury. Previous studies have demonstrated that when infarction involves three or more imaging slices, the risk of END increases significantly (Jang et al., 2020), which is entirely consistent with our finding that the number of infarct slices was markedly higher in the END group than in the non-END group. Maximum infarct area also emerged as a strong predictor of END (Gao et al., 2025). From a pathophysiological perspective, patients with penetrating artery territory infarction have limited collateral circulation, which makes them more susceptible to progressive edema, and lesion extent not only reflects the status of the ischemic penumbra but also correlates with the degree of microcirculatory impairment, with more severe initial ischemic damage associated with a higher likelihood of subsequent deterioration (Liu et al., 2023a). These mechanisms establish a biological basis for using imaging markers of infarct extent to predict END in our model.

In addition to imaging indicators, this study identified LDH as a laboratory marker associated with END. As a sensitive indicator of cellular damage, LDH has been linked to stroke prognosis in multiple studies. Jin et al. (2022) have reported that acute ischemic stroke patients with elevated baseline LDH were more likely to experience early neurological deterioration and had poorer long-term recovery outcomes. In a large cohort of over 5,000 acute ischemic stroke patients, elevated admission LDH was also associated with increased risk of functional disability (He et al., 2024). That study focused on 90-day outcomes rather than early neurological deterioration. Nevertheless, the consistent link between admission LDH and worse stroke outcomes supports its prognostic value across different time windows and treatment settings. The state of ischemia and hypoxia in the brain tissue destroys the membrane structure of neurons, causing LDH to leak into the blood from cells, so the level of LDH can indirectly reflect the size of cerebral ischemia (Jin et al., 2022). It is worth emphasizing that most of the previous studies did not clearly exclude patients who received reperfusion treatment, and this study focused on patients without reperfusion therapy, which could more truly reflect the natural predictive value of LDH. Through SHAP interpretability analysis, we not only verified the positive correlation between LDH level and END risk, but also revealed that its risk contribution accelerated after about 200 U/L. These findings suggest a possibility of clinical threshold. Clinicians who utilize it can quickly identify high-risk patients.

We observed a significant relationship between admission SBP and END. A multicenter randomized ATAMIS study involving 3,005 patients with acute ischemic stroke without reperfusion therapy indicated that higher baseline systolic blood pressure is associated with an elevated risk of early neurological deterioration (Cui et al., 2025). Excessive blood pressure may reduce cerebral perfusion and contribute to neurological deterioration (He et al., 2018). Notably, our SHAP analysis further revealed a non-linear relationship between SBP and END risk, with specific threshold effects that traditional linear models might overlook (Chen et al., 2025; Guo et al., 2025).

The neutrophil count emerged as an inflammatory predictor in our model. Following stroke onset, neutrophils rapidly migrate to the peri-infarct region, where they may amplify edema cascades via the IL-1α/TNF pathways or release neutrophil extracellular traps (NETs) that directly compromise vascular structures, disrupt blood-brain barrier integrity, and impede vascular remodeling as well as reperfusion recovery (Kang et al., 2020; Huang et al., 2024; Sugimoto et al., 2025). Our results show that elevated admission neutrophil levels significantly increase the risk of early deterioration. This is consistent with a multicenter study in acute ischemic stroke, where neutrophils emerged as a key predictor of early neurological deterioration (Zhou et al., 2024). Moreover, both a clinical study focusing on large-vessel occlusion stroke and a large-cohort analysis of inflammatory markers have identified neutrophils as a core independent predictor of poor stroke outcomes (Zhang et al., 2021; Yang Y. et al., 2023). Although the ISRNA study involved patients receiving endovascular therapy (Deng et al., 2021), its finding that elevated inflammatory markers predict early neurological deterioration supports the role of acute-phase inflammation in stroke progression. It should be pointed out that while we evaluated several composite inflammatory indices, including MLR, LMR, NMLR, SIRI, NHHR, and WHR, none demonstrated significant predictive value and were therefore excluded from the final model.

We applied machine learning methods to develop several different prediction models, which improved the stability and clinical practicality of the optimal model. At the beginning of the study, we used LASSO regularization regression to perform variable dimension reduction, which not only avoided multicollinearity in high-dimensional data, but also improved the generalization ability of the model (Yang H. et al., 2023). A retrospective multicenter study confirmed that XGBoost possesses an excellent capacity for capturing non-linear relationships (Livne et al., 2018). This XGBoost model performed stably in the 369 samples of this study, and its robustness in stroke prognosis prediction has been well-documented (Xie et al., 2019). We compared XGBoost with a restricted cubic spline logistic regression model, a simpler non-linear baseline. XGBoost performed better across discrimination, calibration, and clinical utility, indicating that the added complexity yielded tangible gains. Although the DeLong test did not reach statistical significance (*P* = 0.056), likely due to the limited validation set size, XGBoost consistently showed better performance across these multiple metrics, justifying its use over the spline baseline. Nested cross-validation and 1,000-iteration bootstrap internal validation further supported the robustness of XGBoost. Therefore, we chose it as our final modeling algorithm. To overcome the limitation of insufficient interpretability of ordinary machine learning models, we used SHAP analysis to explain and visualize the impact of each indicator on the outcome alone and the impact of multiple indicators together, so as to make the model clear and facilitate an accurate clinical understanding of the source of risk (Lundberg et al., 2020; Huang et al., 2022).

Based on SHAP values, we developed a simple scoring system (0–20 points). The system divides patients into three risk groups. Patients with scores of 0–6 are at low risk and receive routine monitoring. Those with 7–13 points are at intermediate risk and need closer watch plus stronger antithrombotic therapy. Patients with 14–20 points are at high risk and require close monitoring plus aggressive antithrombotic therapy. Recent evidence supports this tiered approach, showing that early intensive therapy reduces END risk in branch atherosclerotic disease (Huang et al., 2025), with high-risk patients potentially benefiting from argatroban addition to dual antiplatelet therapy (Xu et al., 2025). To illustrate how the model could be integrated into clinical workflow, we present the following hypothetical clinical scenario. A 67-year-old male with acute BAD presents with admission SBP of 159 mmHg, LDH of 228 U/L, maximum infarct area of 79.4 mm^2^, two infarct slices, and neutrophil count of 3.86 × 10^9^/L. The scoring system assigns eight points. Three points are given for SBP ≥ 155, five points for LDH ≥ 185, and 0 points each for maximum infarct area < 100, infarct slices < 3, and neutrophils < 4.0, placing him in the intermediate-risk category. Without the model, given his relatively modest infarct size and normal neutrophil count, he might receive only standard care. The model, however, identifies elevated SBP and LDH as combined risk drivers, prompting earlier intervention that could prevent neurological deterioration. This scenario demonstrates how the scoring system helps clinicians quickly assess risk levels at the bedside and formulate corresponding monitoring plans and treatment intensity.

The main innovation of this study lies in the choice of study subjects and the development of the scoring system. As far as we know, this is one of the first studies that looked only at acute BAD patients without reperfusion therapy. In clinical practice, most of these patients are unable to receive such treatment because they are outside the reperfusion time window or have contraindications. Their END risk is significantly different from that of patients who receive reperfusion treatment. However, previous prediction models often put the two groups of patients together for analysis, which has led to the obvious lack of specialized tools for this specific subgroup. In order to meet this clinical need, we specifically excluded patients who had received reperfusion therapy. We used LASSO regression to select variables, built a XGBoost model, and incorporated SHAP interpretability analysis to construct a custom prediction model with good performance and strong practical application. This model provides a precise risk assessment tool for such patients. In addition, we converted the SHAP values into a scoring system. This enables clinicians to quickly perform risk stratification, further improving the model’s clinical usefulness.

In this study, there were several limitations that need to be acknowledged. First, this study was a single-center retrospective study, which cannot completely avoid selection and information bias, and only internal validation was performed. To address this, we plan to carry out a prospective multicenter cohort study in the follow-up to verify the model’s generalizability in different populations and healthcare settings. Second, this model only adopts admission indicators and cannot reflect dynamic changes during hospitalization, including blood pressure, LDH and neutrophil count. Since END develops gradually across days as a progressive clinical course, a prediction model constructed only from baseline admission data may fail to capture key time-related trends that help refine patient risk stratification. A relevant study has indicated that dynamic changes in blood pressure can increase the risk of END (Chung et al., 2015). Therefore, we need to perform dynamic monitoring of these indicators in future research. Third, some risk factors, such as serum neurofilament light chain (Uphaus et al., 2019) and high-sensitive C-reactive protein (Li et al., 2016), were not available in our study. These factors may be potential independent predictors for END. Therefore, it is intended to collect these variables prospectively in our future research. Fourth, although our nested cross-validation and bootstrap validation results support the robustness of our model, we acknowledge that performing feature selection within each fold of the cross-validation loop would provide a more unbiased performance estimate. We plan to adopt this approach in future studies with larger sample sizes. Fifth, the use of machine learning in clinical decision-making raises ethical concerns. This model is intended to serve as a clinical aid rather than replace the judgment of physicians. When deciding on antithrombotic therapy intensity, clinicians should consider this risk score together with their own experience, patient preferences, and the actual clinical situation.

## Conclusion

In summary, we established an XGBoost prediction model and a simple scoring system integrating maximum infarct area, LDH, number of infarct slices, admission SBP, and neutrophil count. SHAP analysis explained how each variable contributes to END risk and revealed their non-linear relationships. This model can predict END risk in acute BAD patients without reperfusion therapy, helping neurologists make individualized antithrombotic decisions at the bedside.

## Data Availability

The raw data supporting the conclusions of this article will be made available by the authors, without undue reservation.

## Supplementary material

Supplementary Figure 1 Calibration curve of the XGBoost model for predicting END in acute BAD patients without reperfusion therapy. The diagonal dashed line indicates perfect calibration. The solid blue curve represents the XGBoost model’s calibration.

Supplementary Figure 2 DCA of the XGBoost model compared with restricted cubic spline logistic regression on the validation set. Decision curve analysis compared the net benefit of four strategies across different threshold probabilities including XGBoost, restricted cubic spline logistic regression, treat all, and treat none. The XGBoost model shown as the red curve outperformed the Spline LR baseline shown as the blue curve with higher net benefit across most thresholds. The gray curve indicated treat all, and the black dashed line indicated treat none.
