# Supplementary material for: Predicting early neurological deterioration in acute branch atheromatous disease without reperfusion therapy: a machine learning model
Source: Front Neurosci. 2026 Jun 10;20:1846221. doi: 10.3389/fnins.2026.1846221 (PMC13290926; doi:10.3389/fnins.2026.1846221)
Supplement: Supplementary file 1 [file Table_1.docx]

**Supplementary Table S1.** **Detailed baseline characteristics of patients with and without END, including composite inflammatory indices and other laboratory parameters.**

| **Baseline characteristics** | **Overall(n=369)** | **Non-END(n=303)** | **END(n=66)** | ***p*** |
| --- | --- | --- | --- | --- |
| **Laboratory data** |  |  |  |  |
| Platelet (×10^9^/L), median (IQR) | 188.00 [157.00, 228.00] | 186.00 [157.00, 226.50] | 203.50 [165.00, 237.00] | 0.228 |
| Hemoglobin (g/L), median (IQR) | 132.00 [120.00, 144.00] | 131.00 [119.00, 143.00] | 137.00 [127.00, 149.75] | 0.003* |
| MPV (fL), median (IQR) | 10.60 [9.80, 11.40] | 10.60 [9.85, 11.50] | 10.50 [9.72, 11.07] | 0.347 |
| RDW-SD (fL), median (IQR) | 42.70 [40.90, 44.70] | 42.90 [40.80, 44.95] | 42.25 [41.00, 43.80] | 0.123 |
| MLR, median (IQR) | 0.26 [0.20, 0.35] | 0.27 [0.21, 0.35] | 0.24 [0.20, 0.35] | 0.195 |
| SIRI, median (IQR) | 0.98 [0.72, 1.48] | 0.95 [0.68, 1.41] | 1.17 [0.82, 1.84] | 0.006* |
| LMR, median (IQR) | 3.79 [2.82, 4.88] | 3.73 [2.83, 4.77] | 4.16 [2.83, 5.07] | 0.199 |
| NMLR, median (IQR) | 2.73 [2.05, 3.79] | 2.66 [1.98, 3.63] | 3.35 [2.40, 4.80] | <0.001* |
| NHHR, median (IQR) | 2.96 [2.22, 3.64] | 2.98 [2.21, 3.62] | 2.84 [2.26, 3.63] | 0.987 |
| WHR, median (IQR) | 4.98 [3.89, 6.32] | 4.91 [3.81, 6.22] | 5.36 [4.36, 7.18] | 0.049* |
| Uric acid (μmol/L), median (IQR) | 316.30 [256.60, 380.70] | 314.30 [258.20, 377.70] | 332.35 [255.98, 394.60] | 0.798 |
| Serum creatinine (μmol/L), median (IQR) | 66.00 [56.00, 80.00] | 67.00 [56.60, 81.75] | 61.80 [51.00, 74.88] | 0.031* |
| Blood urea nitrogen (mmol/L), median (IQR) | 5.20 [4.43, 6.37] | 5.28 [4.49, 6.39] | 5.09 [4.26, 6.08] | 0.148 |
| Estimated glomerular filtration rate (ml/min/1.73m²), median (IQR) | 94.50 [83.89, 102.50] | 93.88 [83.82, 101.80] | 98.25 [87.82, 107.04] | 0.038* |
| Total cholesterol (mmol/L), median (IQR) | 4.80 [4.10, 5.80] | 4.80 [4.10, 5.60] | 5.10 [4.23, 6.18] | 0.039* |
| Triglycerides (mmol/L), median (IQR) | 1.43 [0.99, 2.08] | 1.45 [1.00, 2.08] | 1.42 [0.97, 1.95] | 0.531 |
| HDL-C (mmol/L), median (IQR) | 1.20 [1.04, 1.45] | 1.19 [1.03, 1.42] | 1.31 [1.10, 1.50] | 0.038* |
| LDL-C (mmol/L), median (IQR) | 2.91 [2.19, 3.45] | 2.86 [2.18, 3.40] | 3.09 [2.40, 3.86] | 0.036* |
| D-dimer (mg/L), median (IQR) | 0.62 [0.40, 0.81] | 0.62 [0.40, 0.80] | 0.65 [0.42, 1.00] | 0.301 |
| Fibrinogen (g/L), median (IQR) | 2.84 [2.37, 3.38] | 2.83 [2.36, 3.41] | 2.90 [2.46, 3.24] | 0.848 |
| Infarct location, n (%) |  |  |  | <0.001* |
| Basal ganglia | 67 (18.2) | 51 (16.8) | 16 (24.2) |  |
| Internal capsule | 48 (13.0) | 42 (13.9) | 6 ( 9.1) |  |
| Thalamus | 85 (23.0) | 60 (19.8) | 25 (37.9) |  |
| Pons | 74 (20.1) | 71 (23.4) | 3 ( 4.6) |  |
| Lateral ventricle | 11 ( 3.0) | 11 ( 3.6) | 0 ( 0.0) |  |
| Centrum semiovale | 84 (22.8) | 68 (22.4) | 16 (24.2) |  |
| Stroke side(left), n (%) | 224 (60.7) | 192 (63.4) | 32 (48.5) | 0.035* |
| Onset to door time (hours), median (IQR) | 21.00 [9.00, 30.50] | 23.00 [9.00, 31.25] | 13.75 [6.50, 25.75] | 0.008* |
| Onset to first MRI time (hours), median (IQR) | 23.00 [9.50, 31.50] | 24.50 [9.50, 33.25] | 18.75 [9.12, 26.50] | 0.034* |

**Footnotes:** Values are presented as n (%), mean (SD), or median (interquartile range), as appropriate. Continuous variables were tested for normality by the Shapiro-Wilk test. Normally distributed data were compared using the independent samples t-test, and non-normally distributed data were analyzed using the Wilcoxon rank-sum test. Categorical variables were compared using the χ² test. A P value < 0.05 was considered statistically significant.

**Abbreviations:** MPV, mean platelet volume; RDW-SD, red blood cell distribution width-standard deviation; MLR, monocyte to lymphocyte ratio; LMR, lymphocyte to monocyte ratio; NMLR, neutrophil to monocyte to lymphocyte ratio; SIRI, systemic inflammatory response index; NHHR, non high density lipoprotein cholesterol to high density lipoprotein cholesterol ratio; WHR, white blood cell count to high density lipoprotein cholesterol ratio.

* P < 0.05.
