# Supplementary material for: Predicting early neurological deterioration in acute branch atheromatous disease without reperfusion therapy: a machine learning model
Source: Front Neurosci. 2026 Jun 10;20:1846221. doi: 10.3389/fnins.2026.1846221 (PMC13290926; doi:10.3389/fnins.2026.1846221)
Supplement: Supplementary file 2 [file Table_2.docx]

**Supplementary Table S2. Detailed comparison of clinical data between the training and validation sets, including composite inflammatory indices and other laboratory parameters.**

| **Baseline characteristics** | **Total (n = 369)** | **Training (n = 260)** | **Validation (n = 109)** | ***P*** |
| --- | --- | --- | --- | --- |
| **Laboratory data** |  |  |  |  |
| Platelet (×10^9^/L), median (IQR) | 188.00 [157.00, 228.00] | 189.00 [158.00, 231.00] | 188.00 [152.00, 219.00] | 0.476 |
| Hemoglobin (g/L), median (IQR) | 132.00 [120.00, 144.00] | 133.00 [121.75, 145.00] | 130.00 [120.00, 142.00] | 0.438 |
| MPV (fL), median (IQR) | 10.60 [9.80, 11.40] | 10.60 [9.80, 11.40] | 10.80 [9.80, 11.50] | 0.397 |
| RDW-SD (fL), median (IQR) | 42.70 [40.90, 44.70] | 42.60 [40.60, 44.70] | 43.00 [41.30, 44.80] | 0.243 |
| MLR, median (IQR) | 0.26 [0.20, 0.35] | 0.27 [0.21, 0.35] | 0.26 [0.19, 0.35] | 0.228 |
| SIRI, median (IQR) | 0.98 [0.72, 1.48] | 1.01 [0.73, 1.50] | 0.93 [0.66, 1.41] | 0.298 |
| LMR, median (IQR) | 3.79 [2.82, 4.88] | 3.71 [2.81, 4.75] | 3.82 [2.85, 5.35] | 0.220 |
| NMLR, median (IQR) | 2.73 [2.05, 3.79] | 2.77 [2.04, 3.84] | 2.68 [2.07, 3.76] | 0.558 |
| NHHR, median (IQR) | 2.96 [2.22, 3.64] | 2.95 [2.21, 3.61] | 3.00 [2.24, 3.64] | 0.752 |
| WHR, median (IQR) | 4.98 [3.89, 6.32] | 5.02 [4.00, 6.38] | 4.72 [3.65, 6.27] | 0.635 |
| Uric acid (μmol/L), median (IQR) | 316.30 [256.60, 380.70] | 315.80 [258.30, 375.68] | 319.40 [251.30, 391.60] | 0.540 |
| Serum creatinine (μmol/L), median (IQR) | 66.00 [56.00, 80.00] | 66.00 [54.85, 78.45] | 66.00 [57.00, 83.20] | 0.381 |
| Blood urea nitrogen (mmol/L), median (IQR) | 5.20 [4.43, 6.37] | 5.20 [4.43, 6.35] | 5.36 [4.50, 6.57] | 0.463 |
| Estimated glomerular filtration rate (ml/min/1.73m²), median (IQR) | 94.50 [83.89, 102.50] | 94.71 [84.30, 102.49] | 94.09 [83.50, 102.75] | 0.513 |
| Total cholesterol (mmol/L), median (IQR) | 4.80 [4.10, 5.80] | 4.80 [4.20, 5.70] | 5.00 [4.10, 5.80] | 0.965 |
| Triglycerides (mmol/L), median (IQR) | 1.43 [0.99, 2.08] | 1.40 [1.00, 2.04] | 1.55 [0.99, 2.20] | 0.528 |
| HDL-C (mmol/L), median (IQR) | 1.20 [1.04, 1.45] | 1.20 [1.04, 1.47] | 1.21 [1.04, 1.43] | 0.814 |
| LDL-C (mmol/L), median (IQR) | 2.91 [2.19, 3.45] | 2.91 [2.22, 3.43] | 2.86 [2.12, 3.47] | 0.821 |
| D-dimer (mg/L), median (IQR) | 0.62 [0.40, 0.81] | 0.60 [0.30, 0.80] | 0.70 [0.50, 0.90] | 0.071 |
| Fibrinogen (g/L), median (IQR) | 2.84 [2.37, 3.38] | 2.83 [2.38, 3.38] | 2.92 [2.34, 3.28] | 0.785 |
| Infarct location , n (%) |  |  |  | 0.087 |
| Basal ganglia | 67 (18.2) | 40 (15.4) | 27 (24.8) |  |
| Internal capsule | 48 (13.0) | 30 (11.5) | 18 (16.5) |  |
| Thalamus | 85 (23.0) | 60 (23.1) | 25 (22.9) |  |
| Pons | 74 (20.1) | 57 (21.9) | 17 (15.6) |  |
| Lateral ventricle | 11 ( 3.0) | 7 ( 2.7) | 4 ( 3.7) |  |
| Centrum semiovale | 84 (22.8) | 66 (25.4) | 18 (16.5) |  |
| Stroke side(left), n (%) | 224 (60.7) | 159 (61.2) | 65 (59.6) | 0.876 |
| Onset to door time (hours), median (IQR) | 21.00 [9.00, 30.50] | 24.00 [9.00, 31.00] | 16.00 [9.00, 28.00] | 0.124 |
| Onset to first MRI time (hours), median (IQR) | 23.00 [9.50, 31.50] | 24.50 [9.50, 32.12] | 20.00 [9.50, 30.50] | 0.309 |

**Footnotes:** Values are presented as n (%), mean (SD), or median (interquartile range), as appropriate. Continuous variables were tested for normality by the Shapiro-Wilk test. Normally distributed data were compared using the independent samples t-test, and non-normally distributed data were analyzed using the Wilcoxon rank-sum test. Categorical variables were compared using the χ² test.

**Abbreviations:** MPV, mean platelet volume; RDW-SD, red blood cell distribution width-standard deviation; MLR, monocyte to lymphocyte ratio; LMR, lymphocyte to monocyte ratio; NMLR, neutrophil to monocyte to lymphocyte ratio; SIRI, systemic inflammatory response index; NHHR, non high density lipoprotein cholesterol to high density lipoprotein cholesterol ratio; WHR, white blood cell count to high density lipoprotein cholesterol ratio.

* P < 0.05.
