# Supplementary material for: Predicting early neurological deterioration in acute branch atheromatous disease without reperfusion therapy: a machine learning model
Source: Front Neurosci. 2026 Jun 10;20:1846221. doi: 10.3389/fnins.2026.1846221 (PMC13290926; doi:10.3389/fnins.2026.1846221)
Supplement: Supplementary file 3 [file Table_3.docx]

**Supplementary Table S3. Nested Cross-Validation Results with Enhanced Regularization**

| **Model** | **Nested CV AUC** | **95% CI** | **Mean Gap** | **Max Gap** |
| --- | --- | --- | --- | --- |
| Logistic Regression | 0.871 ± 0.040 | (0.835, 0.940) | 0.006 | 0.053 |
| Decision Tree | 0.815 ± 0.035 | (0.774, 0.858) | 0.069 | 0.123 |
| Random Forest | 0.873 ± 0.037 | (0.840, 0.932) | 0.103 | 0.143 |
| XGBoost | 0.866 ± 0.038 | (0.817, 0.925) | 0.054 | 0.109 |
| LightGBM | 0.870 ± 0.036 | (0.830, 0.924) | 0.115 | 0.168 |
| SVM | 0.872 ± 0.040 | (0.836, 0.941) | 0.003 | 0.055 |
| ANN | 0.865 ± 0.043 | (0.823, 0.938) | 0.023 | 0.091 |

**Abbreviations:** AUC, area under the receiver operating characteristic curve; CI, confidence interval.
