# Supplementary material for: Predicting early neurological deterioration in acute branch atheromatous disease without reperfusion therapy: a machine learning model
Source: Front Neurosci. 2026 Jun 10;20:1846221. doi: 10.3389/fnins.2026.1846221 (PMC13290926; doi:10.3389/fnins.2026.1846221)
Supplement: Supplementary file 4 [file Table_4.docx]

**Supplementary Table S4. Training and validation performance using nested cross‑validation recommended hyperparameters**

| **Model** | **Train AUC** | **Val AUC** | **Gap** |
| --- | --- | --- | --- |
| Logistic | 0.701 | 0.726 | - 0.025 |
| Decision Tree | 0.901 | 0.790 | 0.111 |
| Random Forest | 0.992 | 0.845 | 0.147 |
| XGBoost | 0.927 | 0.839 | 0.087 |
| LightGBM | 0.997 | 0.864 | 0.133 |
| SVM | 0.893 | 0.836 | 0.057 |
| ANN | 0.763 | 0.742 | 0.021 |

**Abbreviations:** AUC, area under the receiver operating characteristic curve; Train, training set; Val, validation set.
