# Supplementary material for: Predicting early neurological deterioration in acute branch atheromatous disease without reperfusion therapy: a machine learning model
Source: Front Neurosci. 2026 Jun 10;20:1846221. doi: 10.3389/fnins.2026.1846221 (PMC13290926; doi:10.3389/fnins.2026.1846221)
Supplement: Supplementary file 5 [file Table_5.docx]

**Supplementary Table S5. Bootstrap Internal Validation of the Final XGBoost Model**

| **Metric** | **Value** |
| --- | --- |
| **Apparent performance** |  |
| Training set AUC | 0.927 |
| Validation set AUC | 0.846 |
| **Bootstrap optimism-corrected performance** |  |
| Bootstrap in-sample AUC (mean ± SD) | 0.970 ± 0.009 |
| OOB AUC (mean ± SD) | 0.855 ± 0.046 |
| OOB AUC 95% CI | (0.760, 0.941) |
| Mean optimism | 0.114 |
| **Agreement with validation set** |  |
| Absolute difference from validation AUC | 0.009 |
| Number of bootstrap iterations | 1000 |
| Successful iterations | 1000 |

**Abbreviations:** OOB, out-of-bag; SD, standard deviation; CI, confidence interval. All bootstrap estimates are based on 1,000 iterations.
