# Supplementary figures and images for: Predicting early neurological deterioration in acute branch atheromatous disease without reperfusion therapy: a machine learning model
Source: Front Neurosci. 2026 Jun 10;20:1846221. doi: 10.3389/fnins.2026.1846221 (PMC13290926; doi:10.3389/fnins.2026.1846221)

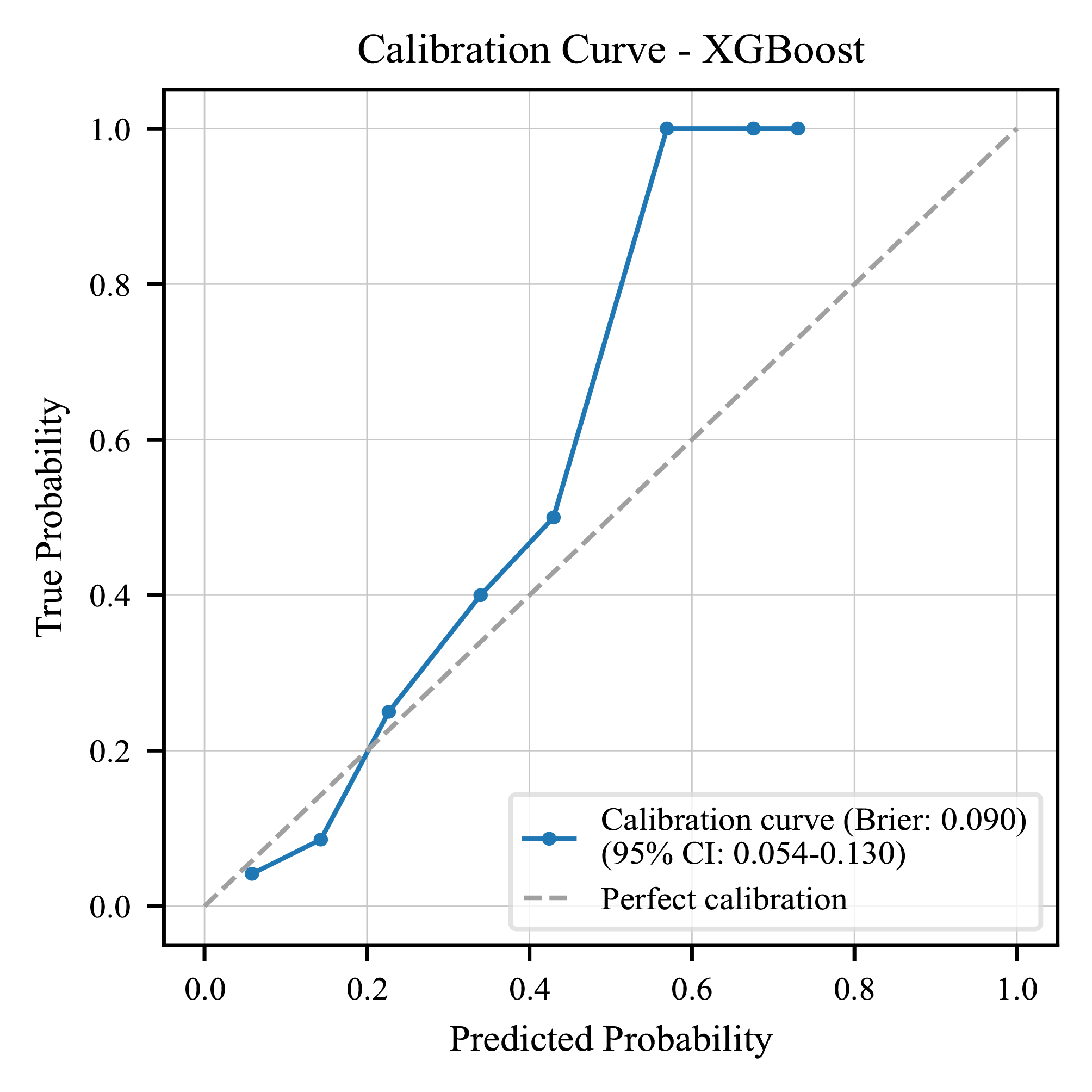

Supplement: Supplementary Figure 1 — Calibration curve of the XGBoost model for predicting END in acute BAD patients without reperfusion therapy. The diagonal dashed line indicates perfect calibration. The solid blue curve represents the XGBoost model’s calibration. [file Image_1.tif]

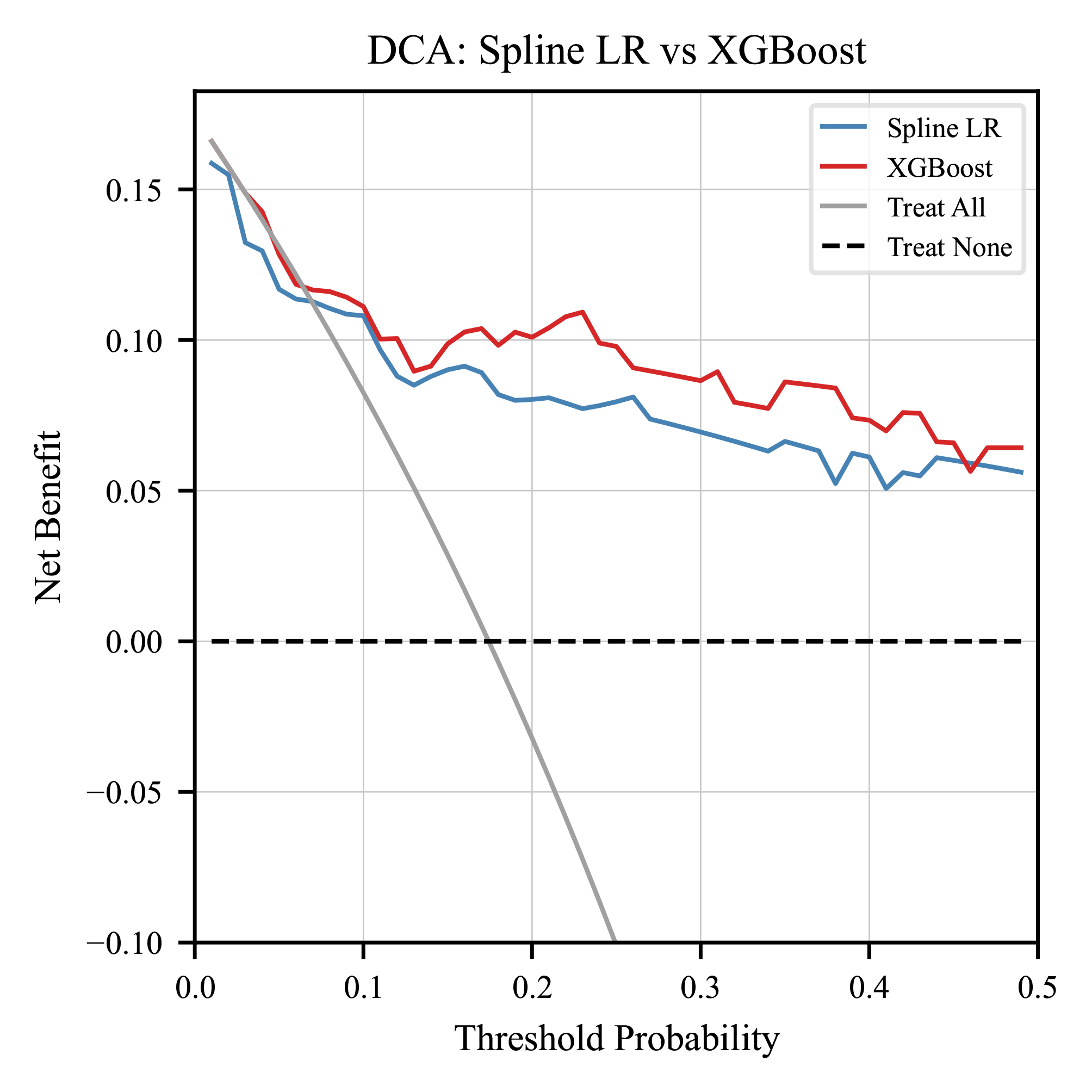

Supplement: Supplementary Figure 2 — DCA of the XGBoost model compared with restricted cubic spline logistic regression on the validation set. Decision curve analysis compared the net benefit of four strategies across different threshold probabilities including XGBoost, restricted cubic spline logistic regression, treat all, and treat none. The XGBoost model shown as the red curve outperformed the Spline LR baseline shown as the blue curve with higher net benefit across most thresholds. The gray curve indicated treat all, and the black dashed line indicated treat none. [file Image_2.tif]
